# Identification and Characterization of a 25 kDa Protein That Is Indispensable for the Efficient Saccharification of *Eisenia bicyclis* in the Digestive Fluid of *Aplysia kurodai*

**DOI:** 10.1371/journal.pone.0170669

**Published:** 2017-01-27

**Authors:** Akihiko Tsuji, Shuji Kuwamura, Akihiro Shirai, Keizo Yuasa

**Affiliations:** 1 Department of Biomolecular function and Technology, Graduate School of Bioscience and Bioindustry, Tokushima University, 2-1 Minamijosanjima, Tokushima, Japan; 2 Department of Bioresource Chemistry and Technology, Graduate School of Bioscience and Bioindustry, Tokushima University, 2-1 Minamijosanjima, Tokushima, Japan; College of Charleston, UNITED STATES

## Abstract

The digestive fluid of the sea hare *Aplysia kurodai* can liberate approximately 2.5 mg of glucose from 10 mg of dried *Eisenia bicyclis* powder. Although laminaran, a major storage polysaccharide in *E*. *bicyclis*, is easily digested to glucose by the synergistic action of the 110 and 210 kDa *A*. *kurodai* β-glucosidases (BGLs), glucose is not liberated from *E*. *bicyclis* by direct incubation with these BGLs. To clarify this discrepancy, we searched for an *Eisenia* hydrolysis enhancing protein (EHEP) in the digestive fluid of *A*. *kurodai*. A novel 25 kDa protein that enhances *E*. *bicyclis* saccharification by β-glucosidases was purified to a homogeneous state from the digestive fluid of *A*. *kurodai*, and its cDNA was cloned from total cDNAs reverse-transcribed from hepatopancreas total RNA. The *E*. *bicyclis* extract strongly inhibited BGLs, suggesting some compound within this brown alga functioned as a feeding deterrent. However, when *E*. *bicyclis* was incubated with BGLs in the presence of EHEP, glucose production was markedly increased. As *E*. *bicyclis* is rich in phlorotannin, which are only found in brown algae, our study suggested that these compounds are the main BGL inhibitors in *E*. *bicyclis* extract. EHEP protects BGLs from phlorotannin inhibition by binding to phlorotannins and forming an insoluble complex with phloroglucinol and phlorotannins. These findings indicated that EHEP plays a key role in the saccharification of brown seaweeds containing phlorotannins in the digestive fluid of *A*. *kurodai*. This is the first report of EHEP as a phlorotannin-binding protein that protects BGLs from inhibition.

## Introduction

Global warming and the depletion of fossil fuels are of great concern, leading to the urgent search for alternative sustainable, renewable, efficient and cost-effective energy sources with reduced greenhouse gas emissions. Biomass seems to be as an excellent alternative source to meet present and future fuel demands. Two of the most common biofuels currently produced are ethanol derived from corn or sugarcane and biodiesel produced from a variety of oil crops, such as soybean and oil perm [1–3]. However the appropriation of significantly higher amounts of corn, sugarcane, or soybean to produce biofuels could have devastating effects on global food availability. To avoid this situation, lignocellulosic feedstocks are being developed as a second-generation of bioethanol sources [4, 5]. However, bioethanol produced from lignocellulosic feedstocks has higher costs, due to the pretreatment required (e.g. steam explosion) to remove lignin and make cellulose accessible for efficient enzymatic digestion [6], and to the larger amount of enzymes needed to produce glucose from cellulosic-based feedstocks than required for starch enzymatic saccharification. Thus, novel technologies for reducing the cost of cellulose saccharification are urgently required [7].

Seaweeds have recently attracted attention as a third-generation biofuel source [7, 8], given their many advantages for renewable energy production: 1), seaweeds are fast growing and photosynthetically more efficient than land plants [9]; 2), most seaweeds lack lignin, which is essential for structural support in terrestrial plants [10] meaning polysaccharide depolymerization in algae is easier than in terrestrial plants [11]; 3), unlike terrestrial plants, seaweeds do not need fresh water, which is increasingly limited in supply; 4), algae can provide sustainable bioremediation of waste-water from a variety of sources through the utilization of growth nutrients such as nitrogen and phosphorous [12]; 5), valuable co-products such as biopolymers, proteins and animal feed can be produced during the fuel generation process [13–15].

Seaweeds (macroalgae) are classified as brown, red, or green algae according to their photosynthetic pigments. Seaweed is utilized in the diets of herbivorous marine animals, such as gastropods and sea urchins. Like terrestrial plants, macroalgae produce many secondary metabolites which play a role in chemical defense against marine herbivores and microbes [16, 17], which include seaweed in their diet. In contrast, herbivores have defense mechanisms for detoxifying and transporting these secondary metabolites [17]. The East-Asian marine gastropod, sea hare (*A*. *kurodai*) consumes seaweed as staple food [18] and, although we have clarified the cellulose [18] and starch digestion systems [19], its defense mechanisms against seaweed secondary metabolites remain unclear. *A*. *kurodai*. possesses two β-glucosidases which showed high amino acid sequence similarity to mammalian intestinal lactase-phlorizin hydrolase, and exhibited β-1, 3, β-1, 4 and β-1, 6-glycoside bond cleavage activities [18]. The two BGLs from the sea hare (210 and 110 kDa enzymes) can produce glucose from laminaran, the major storage polysaccharide of brown algae, consisting of a β-1,3-linked glucose main chain and β-1,6-linked glucose branches [20], without β-1,3-endoglucanases [18].

During our research on the glucose-producing activity of *A*. *kurodai* digestive fluid on *Eisenia bicyclis*, *Ulva pertusa*, *Saccharina sp*., *Undaria pinnatifida* and *Sargassum fusiforme*, we found that *E*. *bicyclis* was the most suitable substrate for glucose production among the seaweed taxa examined. The amount of glucose produced from *E*. *bicyclis* was approximately twice (2.5 mg glucose/10 mg dried seaweed) that liberated by *U*. *pertusa*. However, a minimal amount of glucose was liberated from *E*. *bicyclis* when this alga was incubated with the purified 110 and 210 kDa BGLs found in *A*. *kurodai*, although laminaran was efficiently hydrolyzed to a glucose monomer by the synergistic action of these BGLs. Brown algae such as *E*. *bicyclis*, *E*. *arborea*, *Ecklonia kurome* and *A*. *nodosum*, contain polyphenols called phlorotannins [21–23]. These polyphenols are exclusively found in brown algae and known to deter feeding [24–26], reduce digestibility by inhibiting glycosidases in herbivorous gastropods [27–29]. Therefore, phlorotannins are considered an important component of the chemical defense against marine herbivores. Most phlorotannins are oligomers of phloroglucinol. Eckol (a phloroglucinol trimer), phlorofucoeckol A (a pentamer), dieckol and 8,8’-bieckol (hexamers) were isolated from *E*. *bicyclis* [22]. Thus, phlorotannin might inhibit glucose liberation from *E*. *bicyclis* by the action of 110 and 210 kDa BGLs found in *A*. *kurodai*. Two possibilities might explain why the digestive fluid of *A*. *kurodai* efficiently liberates glucose from *E*.*bicyclis*: 1) the digestive fluid contains special proteins that protect BGLs from an inhibitor; and/or 2) there are other enzymes, besides BGLs, which are capable of liberating glucose and are not inhibited by *E*. *bicyclis*.

The present study aimed to clarify the efficiency of *E*. *bicyclis* digestion by *A*. *kurodai*, by screening the proteins enhancing glucose liberation from *E*. *bicyclis* by incubating this alga with 210 and 110 kDa BGLs and novel laminaran digestive enzymes in the digestive fluid of *A*. *kurodai*. A novel 25 kDa protein which protects BGLs from phlorotannin inhibition, was identified in the digestive fluid of *A*. *kurodai*, and its role in the digestion of *E*. *bicyclis* was investigated.

## Results

### Saccharification Activity of *A*. *kurodai* Digestive Fluid in Various Seaweed Species

**Fig 1:**
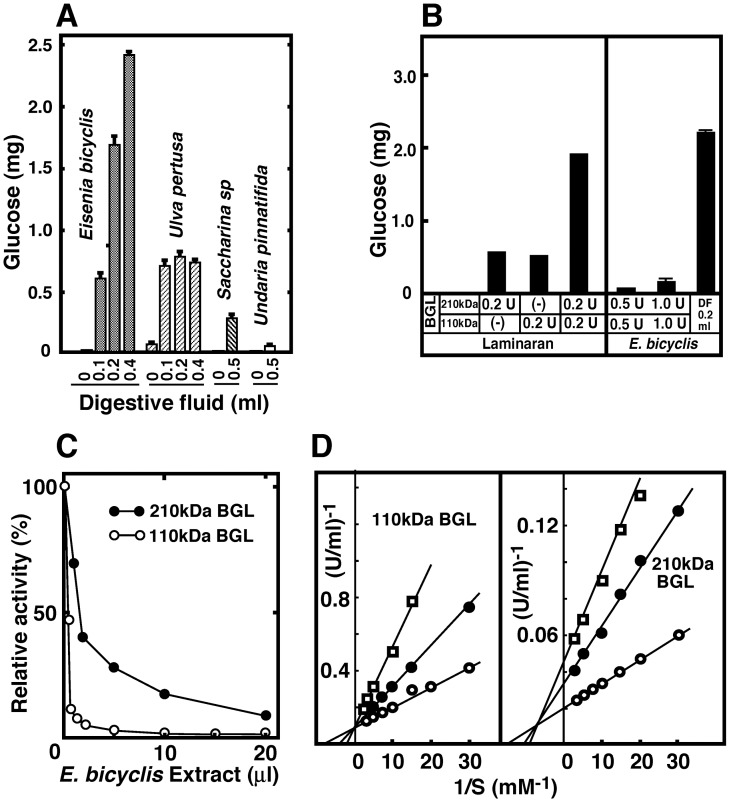
Saccharification of *E*. *bicyclis* by the digestive fluid of sea hare (*A*. *kurodai*) and presence of BGLs inhibitors in the extract of *E*. *bicyclis*. (A) Dried seaweeds (10 mg), *E*. *bicyclis*, *U*. *pertusa*, *Saccharina sp*. and *U*. *pinnatifida* were suspended in 50 mM acetate buffer (pH 5.5) containing 0.1 M NaCl and 10 mM CaCl_2_ (Buffer A), and incubated with several amounts of the digestive fluid (DF) of sea hare at 37°C for 20 h. The glucose content liberated from seaweeds was determined in three independent replicate. (B) Laminaran (2.5 mg) and *E*. *bicyclis* (10 mg) were digested with purified sea hare 110 kDa BGL, 210 kDa BGL, or *A*. *kurodai* digestive fluid at 37°C for 20 h. (C) The activities of 110 and 210 kDa BGLs were assayed in the presence of *E*. *bicyclis* extract. (D) Inhibition mechanism of 110 and 210 kDa BGLs by *E*. *bicyclis* extract. Open circles, extract 0 μl; closed circles, 64-fold diluted extract, 2 μl; open squares, 128-fold diluted extract, 2 μl.

In previous studies, we compared glucose productivity between cellulose and starch digestive systems in various seaweed species using digestive enzymes (endo-β-1,4-glucanases, β-glucosidases, α-amylases and α-glucosidases) purified from *A*. *kurodai* [18, 19]. The amount of glucose liberated from *U*. *pertusa* by the starch digestive system (59 kDa α-amylase + 74 kDa α-glucosidase or 80 kDa α-glucosidase + 74 kDa α-glucosidase) was significantly higher than that liberated by the cellulose digestive system (45 kDa endo-β-1,4-glucanase + 210 kDa BGL). However, none of these digestive systems produced glucose from *E*. *bicyclis*. First, to determine the type of seaweed species that can be easily hydrolyzed to glucose by the digestive fluid of *A*. *kurodai*, dried powder (10 mg) of seven types of seaweed: *E*. *bicyclis*, *U*. *pertusa*, *Saccharina sp*, *U*. *pinnatifida*, *A*. *nodosum*, *Lessonia nigrescens*, and *S*. *fusiforme*, were incubated with the digestive fluid of *A*. *kurodai* (pH 5.5) at 37°C for 24 h and the amount of liberated glucose was determined (Fig 1A).

A minimal amount of glucose was produced from *A*. *nodosum*, *L*. *nigrescens* and *S*. *fusiforme* incubated in the digestive fluid (data not shown). In contrast, *E*. *bicyclis* was the best substrate for *A*. *kurodai* digestive fluid among the seaweed species examined, producing approximately three times more glucose than *U*. *pertusa*. Unlike *U*. *pertusa*, *E*. *bicyclis* contains laminaran, suggesting that this polysaccharide, found in brown algae, might be the major source of the glucose produced by digestive fluid of *A*. *kurodai* acting on *E*. *bicyclis*. Similarly, *E*. *arborea* was efficiently saccharified to glucose by incubation with the digestive fluid of sea hare (data not shown).

### Inhibition of BGL Activity by *E*. *bicyclis* Extract

As shown in Fig 1B, a minimal amount of glucose was produced from *E*. *bicyclis* incubated with purified 210 and 110 kDa BGLs, although purified laminaran was almost hydrolyzed to glucose under the same treatment. Moreover, glucose was not produced from *E*. *bicyclis* incubated with endo-β-1,4-glucanase (45 kDa cellulase) add to both BGLs (data not shown). In the case of *U*. *pertusa*, the amount of glucose liberated by incubation with the digestive fluid of *A*. *kurodai* or with purified cellulolytic and amylolytic enzymes, was almost identical [19].

The brown alga *E*. *bicyclis* contains phlorotannins, which are water-soluble polyphenols [22, 23] that inhibit various glycosidases [27–29]. *E*. *bicyclis* extract inhibited both 110 and 210 kDa BGLs (Fig 1C), but the 110 kDa BGL was more sensitive than the 210 kDa BGL. The modes of inhibition of these enzymes also differed (Fig 1D): competitive inhibition acted upon the 110 kDa BGL, whereas mixed inhibition acted upon the 210 kDa BGL.

**Fig 2:**
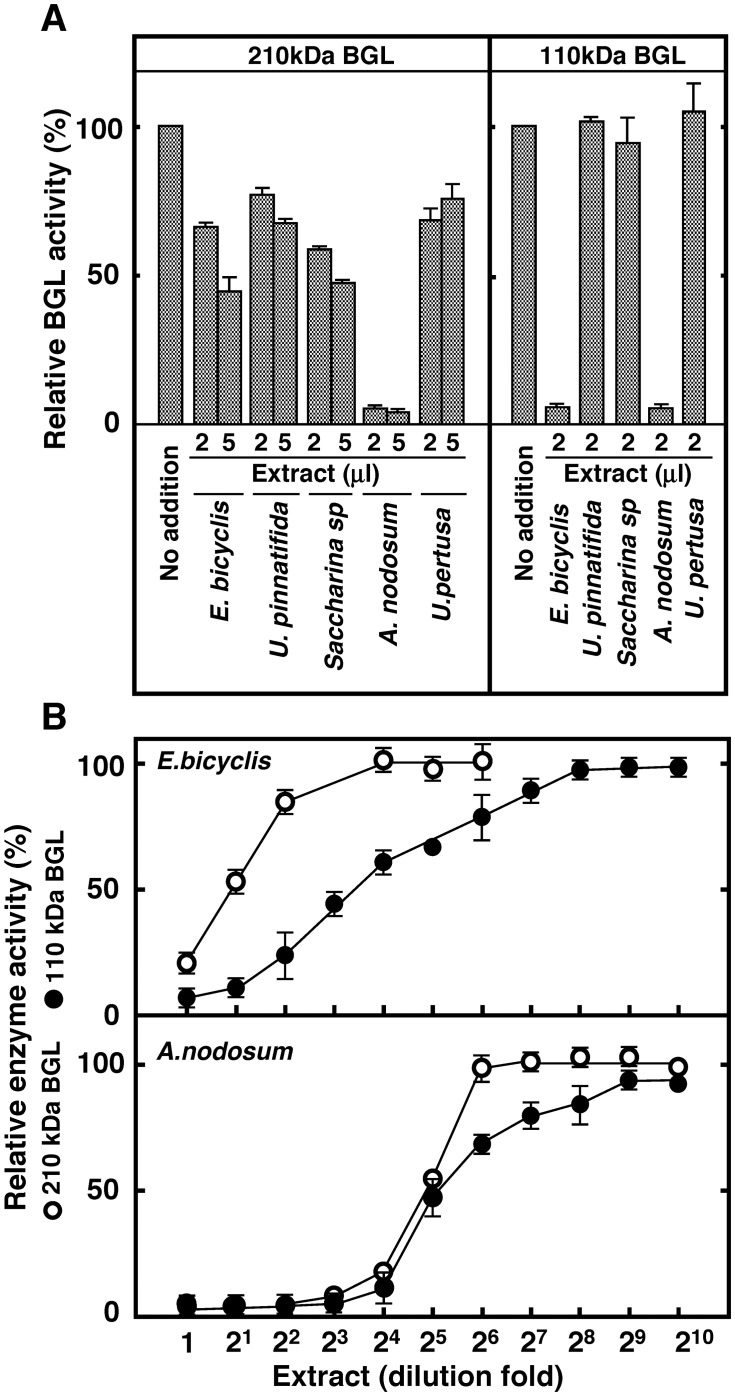
Inhibition of 110 and 210 kDa BGL by extracts of various seaweeds. (A) Ten milligrams of *E*. *bicyclis*, *U*. *pinnatifida*, *Saccharina sp*., *A*. *nodosum*, *and U*. *pertusa* were extracted with 1.0 mL of Buffer A at 4°C for 20 h. After centrifugation, the supernatant (2 or 5 μL) was added into the assay mixture and the activity of each BGL was determined. (B) Inhibitory activity of *E*. *bicyclis* and *A*. *nodosum* extracts against 110 and 210 kDa BGLs. Effect of sequentially diluted extracts on the activity of each BGL was determined. All data (mean ± S.D.) were determined in three independent replicates.

The inhibitory activity of the extracts of several seaweed taxa toward 110 and 210 kDa BGLs were also compared (Fig 2A).

All seaweed extracts except *A*. *nodosum* showed weak inhibitory activities against the 210 kDa BGL. The extract of *A*. *nodosum* had a strong inhibitory activity upon this BGL. Extracts of *E*. *bicyclis* and *A*. *nodosum* strongly inhibited the 110 kDa BGL, whereas other seaweed species had no inhibitory activity against this BGL. Fig 2B compares inhibitory activities of *E*. *bicyclis* and *A*. *nodosum* extract against BGLs. Although both extracts had strong inhibitory activities against the 110 kDa BGL, *A*. *nodosum* inhibition was stronger than *E*. *bicyclis* inhibition of both BGLs, which might be explained by the high amount of phlorotannin present in *A*. *nodosum* [30]. The ethanol extracts of both algae presented similar BGLs inhibition to the water extracts (data not shown). Thus, the inhibition of BGLs, particularly of the 110 kDa enzyme, is likely to be the major reason for the minimal glucose production from *E*. *bicyclis* found in the BGL treatment. These results also suggest the presence of proteins that protect BGLs from phlorotannin inhibition, or of novel laminaran hydrolyzing enzymes, which are not inhibited by phlorotannins, in the digestive fluid of *A*. *kurodai*.

### Purification of *Eisenia* hydrolysis enhancing protein (EHEP) from the Digestive Fluid

**Fig 3:**
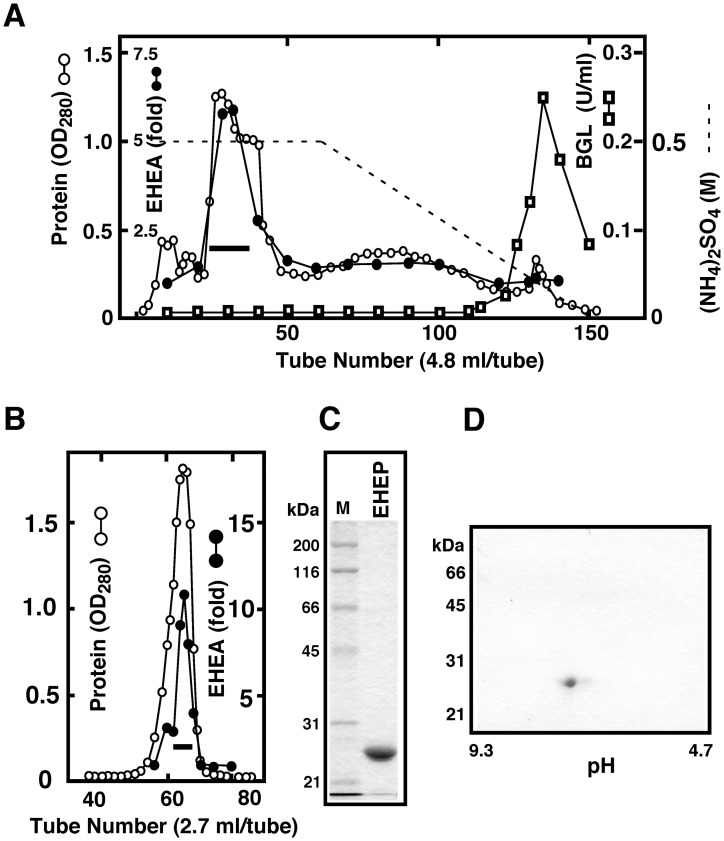
Purification of EHEP from the digestive fluid. The EHEP present in the digestive fluid of the sea hare was purified by ammonium sulfate fractionation (0–30%), phenyl-Sepharose (A), and Sephacryl S-100 gel filtration (B) as described in Materials and Methods. (A) EHEA was eluted in the phenyl-Sepharose unbound fraction. (B) The fraction indicated by the horizontal bar was subjected to Sephacryl S-100 gel filtration. The EHEA fractions indicated by the horizontal bar was concentrated and analyzed by SDS-PAGE (C) and 2D-PAGE (D). Purified EHEP (5 μg) was resolved in both PAGE procedures and detected using Coomassie Brilliant Blue.

Based on the above-mentioned results, we attempted to isolate the proteins protecting BGLs from *E*. *bicyclis* extract inhibition from the digestive fluid of *A*. *kurodai*. The activity of the protective protein EHEP was determined based on the increasing amounts of glucose liberated from *E*. *bicyclis* in the presence of digestive fluid aliquots. The *Eisenia* hydrolysis enhancing activity (EHEA) was found in the ammonium sulfate 0–30% fraction of the digestive fluid, which was further purified by phenyl-Sepharose (Fig 3A) and Sephacryl S-100 (Fig 3B) chromatography, as described in Materials and Methods.

The EHEP was purified to a homogeneous state by sodium dodecyl sulfate (SDS)-polyacrylamide gel electrophoresis (PAGE) and 2D-PAGE (Fig 3C and 3D). The molecular mass of EHEP was estimated as 25 kDa by SDS-PAGE and gel filtration, indicating that EHEP is a monomeric protein. The isoelectric point of EHEP was estimated as 7.0.

### Sequence Analysis of EHEP

In an effort to characterize EHEP at the amino acid sequence level, the N-terminal sequence of the purified EHEP was examined. When an EHEP aliquot > 100 pmol was applied to a protein sequencer, no amino acid sequence was obtained, suggesting that the N-terminus of EHEP is blocked. Consequently, the sequences of EHEP fragments generated by pepsin or lysyl endopeptidase digestion were determined. Three of the internal sequences identified were homologous to chondroitin proteoglycan-2 like proteins sequences, predicted from the *Aplysia californica* genome sequence by automated computational analysis (NCBI reference sequence XP_005103174, XP_005103175 and XP_005103176), particularly to XP_005103174. N-terminal sequences of the pepsin-digested 16 kDa fragment (GNGNFYHPYNCAEYIGCANGLTTVNAXG), pepsin-digested 6 kDa fragment (GFDTYCSANNLATGIHPDPY), and lysyl endopeptidase-digested 21.5 kDa fragment (YISCNGAVATVMXXALGTVFNP) showed 93, 84, and 85% amino acid identities to amino acid residue number 99–126 (**GNGNFYHPYNCA**Q**YI**Q**CANGLTTVNA**C**G**, identical amino acids are in bold), 163–182 (**GFD**S**YCS**V**NNL**SN**GIHPDPY**), and 44–65 (**YI**N**CNGA**I**ATVM**SC**A**P**GTVFNP**) of XP_005103174, respectively.

**Fig 4:**
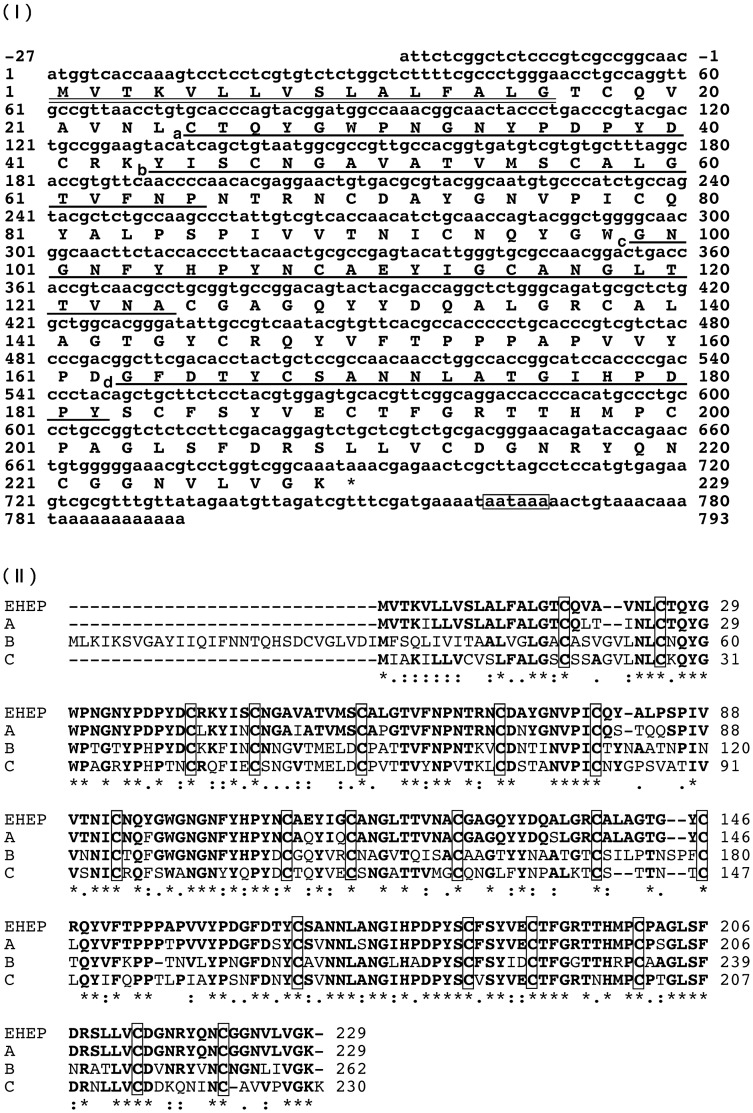
Nucleotide sequence of the EHEP cDNA and the alignment of its deduced amino acid sequence with chondroitin proteoglycan-2-like protein sequences of *A*. *californica* predicted by automated computational analysis. (I) The deduced amino acid sequence is displayed below the nucleotide sequence using a single-letter code. The predicted signal-peptide sequence is double underlined. The amino acid sequences identified by Edman degradation are underlined. The poly-(A) adenylation signal is boxed. (II) A, B, and C sequences are NCBI reference sequences XP_005103174.2, XP_005103176.2 and XP_005103175.1, respectively. Identical amino acid residues among aligned sequences are displayed in bold. The conserved cysteine residues are boxed. The accession number of the nucleotide sequence of EHEP cDNA in the DDBJ/EMBL/GenBank databases is LC122548.

To deduce the primary structure of EHEP, cDNAs encoding the protein were amplified by polymerase chain reaction (PCR) using primers designed from the amino acid sequences of EHEP and the nucleotide sequence of XP_005103174 as described in Materials and Methods. Finally, 5’- and 3’-rapid amplification of cDNA ends (RACE) was performed using specific primers. By combining the nucleotide sequences of these PCR products, a nucleotide sequence of 820 bp was determined (Fig 4-I).

The reliability of the nucleotide sequence was confirmed by PCR using specific primers. The whole nucleotide sequence (DDBJ accession number LC122548) contained a 5’-untranslated region (nucleotide 1–27), an open reading frame (28–717), and 3’-untranslated region (718–820). In the 3’-terminal region, a putative polyadenylation signal (AATAAA, 786–791) and a poly(A) tail (811–820) were found. The amino acid sequences of the fragments produced by pepsin or lysyl endopeptidase digestion matched perfectly with the deduced sequence. The EHEP sequence contained a hydrophobic region close to the initial methionine, which likely corresponds to the signal peptide. The peptide bond between amino acid residues 16 and 17 was predicted to be the cleavage site of the signal peptide using SignalP 4.1 server (www.cbs.dtu.dk/services/SignalP/).

The comparison of the deduced amino acid sequence (total of 229 residues) of EHEP with chondroitin proteoglycan 2-like proteins from *A*. *californica* revealed EHEP’s the highest homology with XP_005103174 (amino acid identity 90%), suggesting that EHEP is an orthologue of XP_005103174 (Fig 4-II). The amino acid identities of EHEP with XP_005103175 and XP_005103176 were 58 and 59%, respectively. The positions of cysteine residues were well conserved among all proteins. The consensus of the conserved cysteines and spacings (CX_15_CX_5_CX_9_CX_12_CX_5-9_C) found in peritrophin A-type chitin binding domains [31] were located in amino acid residues 25–79, 93–146 and 168–221 of EHEP, respectively. These regions of EHEP showed sequence homology to a part of the chitin-binding domain found in antimicrobial chitin binding proteins such as peritrophin-1 [32], peritrophin-95 [33] and tachycitin [34]. The peritrophin-like chitin binding domain containing conserved cysteines has been found in many antimicrobial peptides such as tachycitin [34]. Therefore, the effect of EHEP (50 μg/mL) on the growth curves of *Escherichia coli* and *Staphylococcus aureus* was evaluated as described in Materials and Methods. Antimicrobial activity against both bacteria was not detected (data not shown). Growth curves of *E*. *coli* and *S*. *aureus* were not affected by EHEP.

The chitin binding activity of EHEP was also evaluated as detailed below. EHEP (50 μg) was incubated with chitin fiber (10 mg) in 0.5 mL of Buffer A at 25°C with continuous mixing. After 15 h, the reaction mixture was centrifuged at 12, 000 x *g* for 5 min. The precipitate was treated with SDS-PAGE loading buffer and analyzed by SDS-PAGE. The binding of EHEP with chitin fiber and a decrease of EHEP in the supernatant were not detected. These results indicated that EHEP does not present antimicrobial and chitin binding activities.

### Role of EHEP in Seaweed Digestion

**Fig 5:**
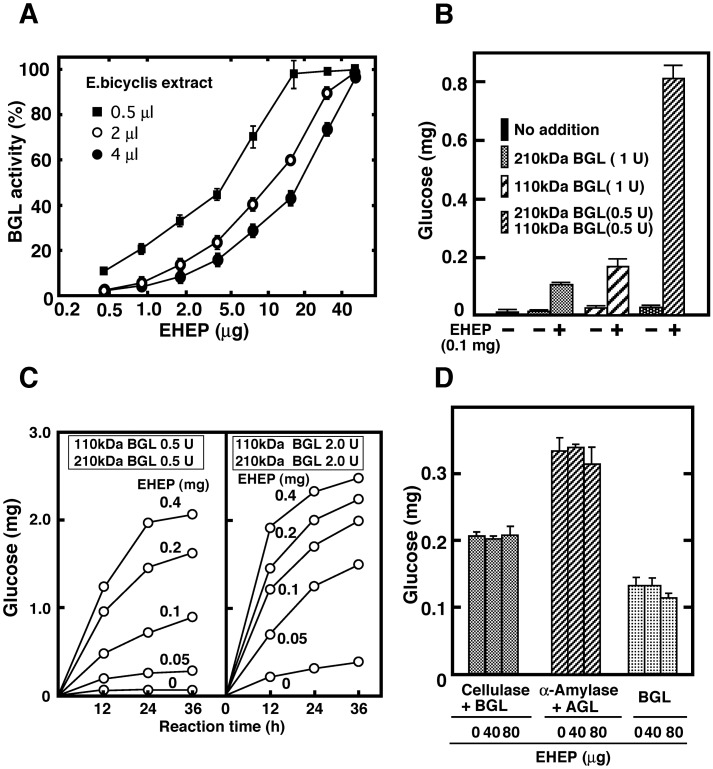
EHEP protects BGL activity from *E*. *bicyclis* extract inhibition. (A) The 110 kDa BGL activity was assayed in a reaction mixture containing 1 mM 4MU-β-glucoside, *E*. *bicyclis* extract (0.5, 2.0, or 4.0 μL), and EHEP. The enzyme activity (mean ± S.D.) was determined in three independent replicates. (B) *E*. *bicyclis* (10 mg) was suspended in 1.0 mL Buffer A, and incubated with 210 or 110 kDa BGL in the absence or presence of EHEP at 37°C for 6 h. Glucose released was determined. (C) Saccharification of *E*. *bicyclis* by the synergistic action of 110 and 210 kDa BGLs in the presence of EHEP. *E*. *bicyclis* (10 mg) was incubated with a mixture of 110 and 210 kDa BGL in the absence or presence of EHEP in 1.0 mL Buffer A at 37°C for 24 h. Glucose (mean ± S.D.) was determined in at least three independent replicates. (D) *U*. *pertusa* (10 mg) was incubated with 45 kDa cellulase (0.5 U), 59 kDa α-amylase (0.5 U), 74 kDa α-glucosidase (AGL, 0.2 U), 210kDa BGL (0.4 U) and 110 kDa BGL (0.2 U) in the absence or presence of EHEP in 1.0 mL of Buffer A at 37°C for 24 h. Glucose (mean ± S.D.) was determined in three independent replicates.

As shown in Figs 1C and 2, the *A*. *kurodi* 110 kDa BGL was completely inhibited by *E*. *bicyclis* extract. We examined the effect of EHEP on *E*. *bicyclis* extract inhibition of 110 kDa BGL. Fig 5A shows the dose-dependent EHEP protection of 110 kDa BGL inhibition by *E*. *bicyclis* extract.

The 110 kDa BGL activity was completely protected from inhibition by *E*. *bicyclis* extract (2 μL) inhibition by 50 μg EHEP. Although the 210 kDa BGL was less sensitive to the extract, its activity was also increased by the addition of EHEP (data not shown). Fig 5B shows the effect of EHEP on glucose production from *E*. *bicyclis* by digestion with 110 and 210 kDa BGLs. When 110 or 210 kDa BGLs were incubated with *E*. *bicyclis* in the absence of EHEP, glucose was hardly released but significantly increased in the presence of EHEP. Fig 5B also shows the synergistic effect of 110 and 210 kDa BGLs in the presence of EHEP on the production of glucose from *E*. *bicyclis*. When *E*. *bicyclis* (10 mg) was digested with one unit of 110 or 210 kDa BGL in the presence of 0.1 mg/mL of EHEP at 37°C for 48 h, the total glucose released from *E*. *bicyclis* in each reaction was 0.16 and 0.11 mg, respectively. However, when *E*. *bicyclis* was digested with a mixture of 110 (0.5 U) and 210 kDa (0.5 U) BGLs in the presence of EHEP, there was an approximately five to seven-fold increase in glucose production (0.81 mg). Glucose production from *E*. *bicyclis* by digestion with a mixture of both BGLs in the presence of different concentrations of EHEP was also examined (Fig 5C). The enzyme activities of 110 and 210 kDa BGLs in the digestive fluid of *A*. *kurodai* were 2.0–4.0 U/mL. As shown in Fig 5C, the glucose production from *E*. *bicyclis* increased with increasing doses of EHEP for similar BGLs activities in the digestive fluid of *A*. *kurodai*. Approximately 2.5 mg of glucose were produced from 10 mg of dried *E*.*bicyclis* in the digestive fluid (Fig 1A and 1B) incubated with 1.0 U total activity of BGL in the presence of 0.4 mg/mL EHEP, or 4.0 U of BGLs in the presence of 0.2–0.4 mg/mL of EHEP for 36 h. These results indicate that EHEP is an essential protein for efficient saccharification of *E*. *bicyclis* by both 210 kDa and 110 kDa BGLs.

We also examined if EHEP enhances the saccharification of the sea lettuce, *U*. *pertusa*, as the sea hare individuals inhabiting the Naruto coast showed a clear preference for this seaweed. As shown in Fig 5D, EHEP had no effect on saccharification of *U*. *pertusa* by purified glycosidases. These results strongly suggest that EHEP increases glucose production from laminaran digestion by BGLs in *E*. *bicyclis*.

### Binding of EHEP with Phlorotannin

**Fig 6:**
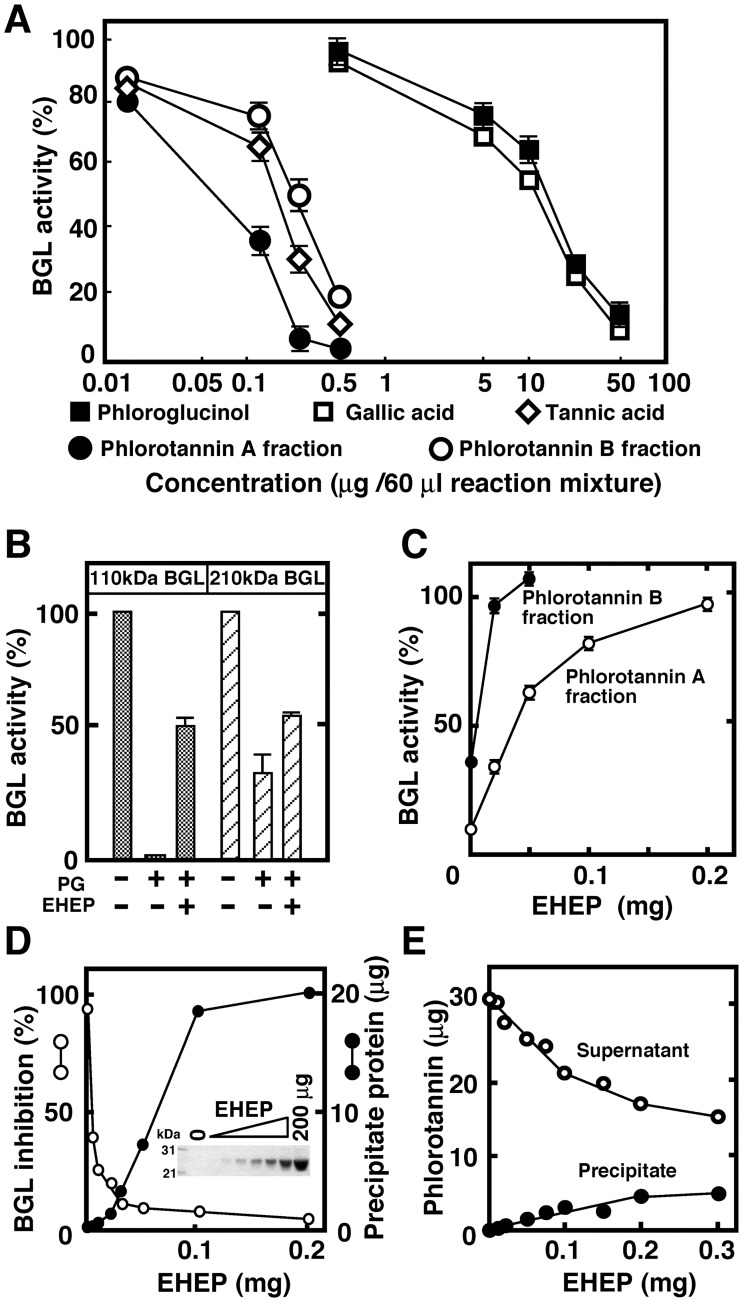
Interaction of EHEP with phlorotannin, tannic acid, phloroglucinol, and gallic acid. (A) Inhibition of 110 kDa BGL by phlorotannin, tannic acid, phloroglucinol and gallic acid. The activity of the 110 kDa BGL was assayed in a reaction mixture containing phlorotannin A (diethyl ether fraction), phlorotannin B (aqueous fraction), tannic acid or phloroglucinol at the indicated concentrations. (B) Effect of EHEP on the inhibition of BGLs by phloroglucinol. The activities of the 110 and 210 kDa BGLs were assayed in the absence and presence of 2 mM phloroglucinol (PG) and EHEP (0.28 mg/mL). (C) Protection excerted by EHEP upon BGL’s inhibition by phlorotannin. The activities of 210 and 110 kDa BGLs were assayed in a reaction mixture containing 0.5 μg phlorotannin A in the absence and presence of the indicated EHEP concentration. (D) Reverse-correlation of EHEP precipitation and BGL inhibition by *E*. *bicyclis* extract. *E*. *bicyclis* extract (10 μL) was incubated with EHEP in 0.1 mL Buffer A at 25°C for 1 h. The precipitate formed was washed with the same buffer, and protein contents and composition were analyzed. The inhibitory activity of the supernatant was assayed. SDS-PAGE of precipitated proteins is shown in the inset. (E) Precipitation of *E*. *bicyclis* phlorotannins by EHEP. *E*. *bicyclis* extract (0.1 mL; 100 mg *E*. *bicyclis* /1.0 mL of Buffer A) was incubated with the indicated EHEP concentrations at 4°C for 16 h. After centrifugation at 12, 000 x *g* for 10 min, the supernatant and the precipitate were separated. The precipitate was washed twice with Buffer A and suspended in the same buffer. The content of phlorotannin in the supernatant and precipitate were determined as described in Materials and Methods.

Phlorotannins are found in most brown algae, including *E*. *bicyclis*, *Ecklonia cava* and *A*. *nodosum* [21–23, 30]. As shown in Fig 2A, *E*. *bicyclis* and *A*. *nodosum* extracts strongly inhibited the 110 kDa BGL whereas other brown seaweed (*U*. *pinnatifida* and *Saccharina sp*) did not. Phlorotannin contents in brown seaweed species differ: *E*. *bicyclis*, *E*. *cava*, and *A*. *nodosum* are rich sources of phlorotannin, whereas *U*. *pinntifida* has low phlorotannin content [35]. Thus, the inhibitory effect of seaweed extracts on the activities of BGL appears to be associated with their phlorotannin content. These results strongly suggest that phlorotannin is a potential candidate for a BGL inhibitor. Given that phlorotannins are oligomers of phloroglucinol (1,3,5-trihydroxybenzene), which are classified into polyphenol compounds, the inhibition of BGLs by phloroglucinol, gallic acid, tannic acid, and phlorotannins isolated from *E*. *bicyclis* were compared (Fig 6A).

Phlorotannin and tannic acid comprise phloroglucinol and gallic acid, respectively. Phlorotannins were fractionated from the ethanol extract of *E*. *bicyclis* using diethyl ether (A fraction) and water fraction (B fraction), as described in Materials and Methods. The inhibitory activity of A fraction (Phlorotannin A) of phlorotannin was slightly stronger than that of the B fraction (Phlorotannin B), but both presented lower IC_50_ (inhibitor concentration required for 50% inhibition) than phloroglucinol and gallic acid (about 100-fold higher in these phenol compounds than in phlorotannin).

As shown in Fig 6B, phloroglucinol inhibited both 110 and 210 kDa BGLs, and similar to that observed for *E*. *bicyclis* extract, 110 kDa BGL was more sensitive to phloroglucinol than 210 kDa BGL. The inhibition of BGL by phloroglucinol was partially protected (approximately 50%) by the addition of EHEP. In contrast, the inhibition of BGL by phlorotannins was totally protected by EHEP (Fig 6C). After incubating EHEP with phlorotannin, BGL activity in the supernatant and phlorotannins content in the precipitate were examined (Fig 6D). During incubation, EHEP was precipitated and the inhibitory activity of phlorotannins over BGL was lost. We then examined the effect of EHEP on the phlorotannin contents of the *E*. *bicyclis* extract, by incubating the extract with EHEP. After incubation, phlorotannin contents in the supernatant and precipitate were determined. As shown in Fig 6E, the phlorotannin content in the supernatant was reduced by the precipitation of EHEP whereas phlorotannin content in the precipitate increased. Thus, EHEP is able to bind to phlorotannin in purified and crude systems.

**Fig 7:**
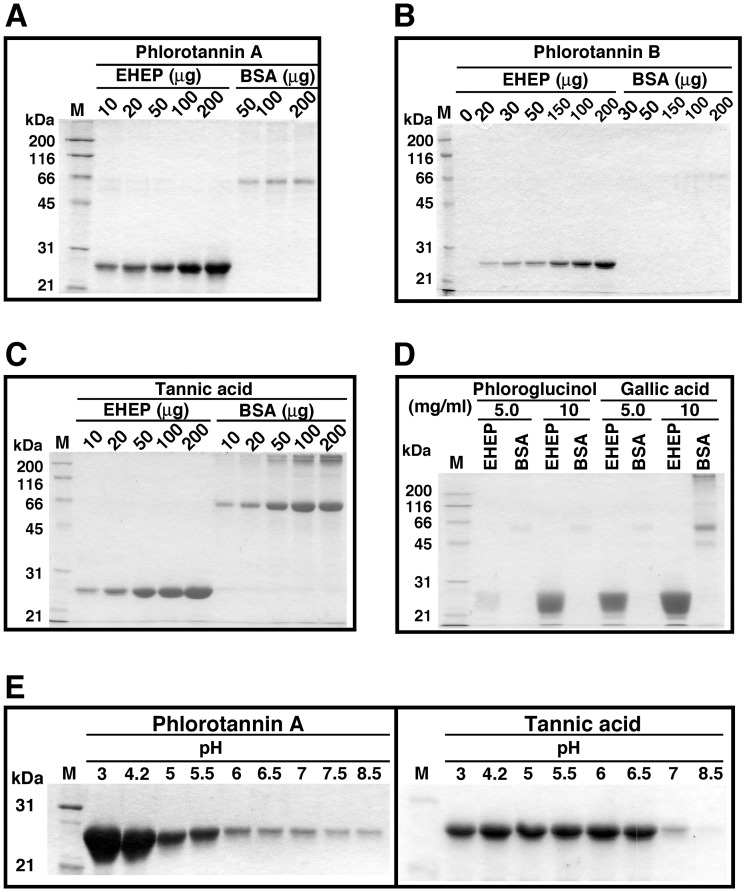
Specific precipitation of EHEP with phlorotannins. Phlorotannin fractions A and B (10 μg of phloroglucinol equivalent) isolated from *E*. *bicyclis*, phloroglucinol (50 and 100 μg) or gallic acid (50 and 100 μg) were incubated with EHEP or BSA in 0.1 ml Buffer A at 25°C for 90 min. The reaction mixture was centrifuged at 12, 000 x *g* for 10 min. The resulting precipitate was dissolved in SDS-PAGE loading buffer containing 1% β-mercaptoethanol, treated at 95°C for 5 min and applied to 12% SDS-PAGE. Protein was detected by Coomassie Brilliant Blue staining. Precipitation of BSA with polyphenols was used as control, given that BSA is a polyphenol binding protein.

Fig 7 shows the SDS-PAGE analysis of EHEP precipitation after its incubation with phlorotannins, tannic acid, phloroglucinol and gallic acid.

BSA binds to several polyphenol compounds [36]. EHEP was precipitated by phlorotannin A and phlorotannin B in a concentration–dependent mode (Fig 7A and 7B), and it was more efficiently precipitated with phlorotannin A than with phlorotannin B. In contrast, BSA was hardly precipitated by incubation with either phlorotannin: a minimal amount of precipitate was detected when BSA was incubated with phlorotannin A. Although EHEP and BSA were similarly precipitated by tannic acid (Fig 7C), EHEP was precipitated by phloroglucinol and gallic acid (even more reactive; Fig 7D), but BSA was hardly precipitated by these compounds. Only a trace amount of BSA was precipitated when this protein was incubated with 10 mg/mL gallic acid. EHEP binding to phlorotatnnins and phloroglucinol seems to be more efficient than that of BSA.

The effect of pH on EHEP precipitation by phlorotannins and tannic acid was also examined (Fig 7E), revealing that EHEP was efficiently precipitated by phlorotannin in a pH-dependent manner, particularly at acidic pH. In contrast, EHEP precipitation by tannic acid did not show a pH-dependence at pH 3–6.5, although EHEP was not precipitated at a higher pH than pH 7.0.

### Screening of a Novel Laminaran Digestive Enzyme

To isolate a novel laminaran digestive enzyme, we screened the β-1,3-glycoside bond cleaving activity in the digestive fluid using curdlan as a substrate. As curdlan is a β-glucan consisting of a single β-1,3-linkage, it was possible to purify curdlan digestive enzyme (CDE), which is larger than known laminaranases [37], to a homogenous state (S1 Fig). Using a NaCl gradient on Mono S chromatography (S1A Fig), CDE was separated from the 110 kDa BGL and eluted as a single protein and its molecular mass was estimated as 85 kDa by SDS-PAGE (S1B Fig). Internal sequences of CDE were homologous to regions of the α-xylosidase A-like protein (NCBI reference sequence: XP_012938183) predicted from the genome of *A*. *californica*, α-xylosidase (WP_039080918) from *Gallibacterium anatis*, glucan 1,3-α-glucosidase (XP_011667610) from *Stronglylocentrotus purpuratus*, and α-glucosidase (WP_003507443) from *Clostridium symbiosum* (S1C Fig). In particular, all three internal sequences of CDE were highly homologous with the region of α-xylosidase A-like protein from *A*. *californica*. Lectin blot results suggested CDE is a glycoprotein (S1D Fig), as it reacted with concanavalin-A and LCA lectins, and that it contains a high-mannose type oligosaccharide. These results also indicated that CDE is not derived from gut bacteria.

Although CDE hydrolyzed curdlan and laminarin to glucose, maltose and glucotriose (S2A Fig), it was CDE is more active toward curdlan than toward laminaran. When curdlan was incubated with CDE and 210 kDa BGL, maltose and glucotriose were converted to glucose. As shown in S2B Fig, glucose production from laminaran by BGLs was increased by the addition of CDE and there was a particular synergistic effect of 210 kDa BGL and CDE. However, when the synergistic effect of both BGLs and CDE on glucose production from laminaran was examined, there was no significant effect on glucose production from laminaran when CDE was used in conjugation with the 210 or 110 kDa BGLs (S2C Fig). These results suggested that *E*. *bicyclis* saccharification in the gut is mainly performed by combination of 210 and 110 kDa BGLs in the presence of EHEP, although laminaranases and CDE are likely to assist the laminaran digestion performed by both BGLs.

## Discussion

The most striking finding in the present investigation was the identification of a novel 25 kDa EHEP, which binds to polyphenols in brown seaweed. To survive against predation by marine animals such as sea urchin and gastropods, seaweeds produce feeding deterrents [16, 17], such as the phlorotanins of *E*. *bicyclis* that have been shown to have a deterrent effect against the feeding behavior of the herbivorous gastropod *Turbo cornuts* [26]. In the present study, we showed that *E*. *bicyclis* extract contained a strong inhibitor of *A*. *kurodai* β-glucosidases, particularly the 110 kDa BGL, which was highly sensitive to *E*. *bicyclis* and *A*. *nodosum* extracts. Although purified laminaran was efficiently digested to glucose by the 110 and 210 kDa BGLs, saccharification of *E*. *bicyclis* laminaran by these BGLs was almost completely blocked by *E*. *bicyclis* compounds; however the inhibitory activity of the *E*. *bicyclis* extract was completely neutralized with EHEP. *E*. *bicyclis* contains several kinds of phlorotannins, eckol (phloroglucinol trimer), phlorofucoeckol A (pentamer), dieckol and 8,8’-bieckol (hexamers) [22, 23, 38]. Although we did not identify individual phlorotannin-inhibiting β-glucosidases, several results suggested phlorotannins as the most plausible candidates for BGL inhibitors: 1) BGLs were inhibited in brown seaweed extracts containing a high content of phlorotannins, as is the case of *E*. *bicyclis* and *A*. *nodosum*, and extracts of the green seaweed, *U*. *pertusa* had no effect on β-glucosidase activity; 2) the phlorotannin fraction isolated from the 80% ethanol extract of *E*. *bicyclis* (phlorotannin A) strongly inhibited BGLs, but was neutralized when incubated with EHEP, and EHEP was precipitated by phlorotannins; 3) phloroglucinol, a constituent of phlorotannins, also inhibited BGL, and EHEP was precipitated by phloroglucinol, although a higher concentration of phloroglucinol was required for BGL inhibition. In addition, the affinities of EHEP toward phlorotannins and phloroglucinol were higher than that of BSA, EHEP and BSA had similar affinities to tannic acid, and EHEP was more specific toward phlorotannins comprising phloroglucinol than for tannin comprising gallic acid.

A relatively high concentration of EHEP (> 0.1 mg/mL) was required for efficient saccharification of *E*. *bicyclis* by the 210 and 110 kDa BGLs. Given that approximately 20 mg of EHEP were purified from 200 mL of *A*. *kurodai* digestive fluid, the concentration of EHEP in the digestive fluid is expected to be > 0.1 mg/mL. It is highly likely that the concentration of EHEP in the digestive fluid is sufficient to suppress the inhibitory activity of phlorotannin against BGLs. Thus, these results indicate that EHEP plays a critical role in *E*. *bicyclis* saccharification in the digestive fluid, similar to the *in vitro* digestion performed by the two β-glucosidases in the presence of EHEP.

To date, two families of salivary tannin binding proteins, proline-rich proteins and histatins, have been identified in mammals, and their physiological roles have been proposed [39–41]. Tannins are polyphenols found in terrestrial plants, and tannin-binding proteins deactivate tannins by forming complexes with them, thereby preventing their interaction with other biological compounds and their absorption in the intestinal canal. Although BSA is known to possess polyphenol binding activity [36], the affinity of proline-rich proteins to tannins is higher than that of other proteins. Various harmful effects of dietary tannins such as growth retardation, perturbation of mineral absorption, and inhibition of digestive enzymes were reported [40], and salivary proline-rich proteins and histatins are likely to form complexes with tannins from food, thereby minimizing their harmful effects. It is highly likely that EHEP also minimizes the harmful effect of phlorotannins in brown algae, including glycosidases inhibition.

However, the amino acid sequences of EHEP, proline-rich proteins, and histatins are not homologous. The amino acid composition of salivary proline-rich proteins is unique in containing remarkably high proline contents, although glycine, glutamic acid and glutamine contents are also high. Histatins are a group of relatively small proteins with a high affinity to tannins [40, 41], only found in humans and some primates, and characterized by a high content of histidine. On the other hand, EHEP is characterized by a high content of asparagine (21 residues), followed by cysteine (19 residues), tyrosine (19 residues) and proline (17 residues). BLAST search showed that EHEP is a unique protein, as no proteins or predicted gene products with homologous amino acid sequences and molecular sizes similar to that of EHEP are found in other organisms. As a notable sequential feature, EHEP has a consensus of conserved cysteines and spacings (CX_15_CX_5_CX_9_CX_12_CX_5-9_C) found in peritrophin A-type chitin-binding domains of amino acid residues 25–79, 93–146 and 168–221[31]. These consensus cysteine sequences are also found in EHEP homologues from *A*. *californica*. Proteins with a peritrophin A-type domain show chitin binding and antimicrobial activities against Gram positive and Gram negative bacteria [32–34], and are suggested to play a role in the spatial organization of the peritrophic matrix. However, the complete amino acid sequence of EHEP is not related to any of these proteins, and no chitin-binding and antimicrobial activities were detected in EHEP. Thus, the function of the conserved cysteine residues in EHEP remains unknown.

In conclusion, the present study showed that a 25 kDa phlorotannin-binding protein, EHEP, protects BGLs from *E*. *bicyclis* phlorotannin inhibition and accelerates saccharification of laminaran in the alga. The results of the present study provide important insight concerning the *A*. *kurodai* defense mechanisms against feeding deterrents, such as phlorotannins from *E*. *bicyclis*. Regarding biofuel production, the present study constitutes an important step in our effort to identify a digestion system that can release glucose from *E*.*bicyclis*.

## Materials and Methods

### Materials

*A*. *kurodai* (body length, 20–25 cm), *U*. *pertusa* (sea lettuce) and *U*. *pinnatifida* (wakame) were collected along the coast of Naruto, Japan, from April to July. These three species are not protected in this area, and therefore, no specific permissions were required for their collection. After dissection, the digestive fluid of *A*. *kurodai* was obtained from the gastric lumen by squeezing the stomach and stored at -30°C until further use [18]. *U*. *pertusa* and *U*. *pinnatifida* were washed with water, dried at 50°C, and then pulverized in a Waring blender. Dry powdered *A*. *nodosum* was purchased from Starwest Botanicals, Inc. (Sacramento, CA, USA) and dry powdered *Lessonia nigrescens* collected in Chile was purchased from Michinoku Farm (Saitama, Japan). Dried *E*. *bicyclis* collected from Nakamura coastal area (depth 2–5 m; Oki Island, Shimane prefecture, Japan), during all seasons, *E*. *arborea* collected from Ise-shima area (Wakayama prefecture, Japan), in the summer, *S*. *fusiforme* collected in Korea in early spring, and *Saccharina sp*. collected from the coast of East-Hokkaido (Japan), in the summer, were purchased from a local grocery store and pulverized in a Waring blender. *E*. *bicyclis*, *E*. *arborea and S*. *fusiforme* were sun-dried and soaked in seawater for 24 h. After this period, these seaweeds were washed in water, boiled and sun-dried before further use. *Saccharina sp*. was only sun-dried before use.

Corn starch, D-(+)-glucose, laminaran from *E*. *bicyclis*, phloroglucinol and the Glucose CII Test Wako were purchased from Wako Pure Chemicals (Osaka, Japan). 4-Methylumbelliferyl (4MU)-α-glucoside and 4-methylmbelliferyl (4-MU)-β-glucoside were from Sigma-Aldrich (St, Louis, MO, USA). A peroxidase-labeled lectin kit was purchased from J-OIL MILLS (Tokyo, Japan). Phenyl-Separose, Sephacryl S-100, and Mono-S HR5/5 were obtained from GE Healthcare (Uppsala, Sweden). All other chemicals used were of analytical grade.

The α-Amylase (59 kDa), α-glucosidases (74 and 84 kDa), BGLs (110 and 210 kDa), β-1,4-endoglucanases (21 and 45 kDa), and β-1,3-endoglucanase (laminaranase) were purified from the digestive fluid of *A*.*kurodai* to the homogeneous state, as previously described [18, 19].

### Enzyme Assay

*Eisenia* hydrolysis enhancing activity (EHEA) was assayed by measuring the increase in glucose release from *E*.*bicyclis* after incubation with EHEP and BGLs. The reaction mixture (1.0 mL) consisted of 10 mg dried *E*. *bicyclis* powder, 50 mM acetate buffer (pH 5.5), 0.1 M NaCl, 10 mM CaCl_2_, 110 kDa BGL (0.5 U), 210 kDa BGL (0.5 U), and an appropriate amount of sample. Following incubation at 37°C for 18 h, the reaction was terminated by heat treatment at 95°C for 5 min. The amounts of glucose liberated by *E*.*bicyclis* saccharification was then determined using the Glucose CII Test Wako kit. The activities of BGL and α-glucosidase were assayed using 4-MU-β-glucoside and 4-MU-α-glucoside, respectively, as previously described [42], and the released 4-methylumbelliferone was measured fluorometrically (excitation, 365 nm; emission, 450 nm). The activity producing 1 μmol of 4-methylumbelliferone per min at 37°C was defined as one unit (1 U). The activity of the curdlan digestion enzyme (CDE) activity was assayed in a 0.2 mL reaction mixture containing 1% curdlan, 50 mM acetate buffer (pH 5.5) and an appropriate amount of enzyme at 37°C. Following incubation for 10–60 min, the reaction was terminated by heat treatment (95°C, 2 min). The reducing sugar liberated by curdlan hydrolysis was determined by the method of Nelson and Somogyi [43]. The activity liberating an amount of reducing sugars equivalent to 1 μmol of glucose per min was defined as 1 U. Protein concentration was determined by the Bradford method using BSA as the standard [44].

### Analysis of *E*. *bicyclis* Inhibition Toward 110 and 210 kDa BGLs

Dried *E*. *bicyclis* (40 g) was extracted with 100 mL of 50 mM acetate buffer (pH 5.5), containing 0.1 M NaCl and 10 mM CaCl_2_ (Buffer A), at 4°C for 16 h, with continuous stirring. After centrifugation, the supernatant was used to assess the mechanism by which the *E*.*bicyclis* extract inhibited BGLs, using Line-weaver Burk plot method. The activity of the 110 kDa BGL toward different concentrations of 4MU-β-glucoside was assayed in the absence and presence of 64- and 128-fold diluted *E*. *bicyclis* extract (2 μL), whereas the activity of the 210 kDa BGL was assayed in the absence and presence of non-diluted *E*.*bicyclis* extract (2 and 4 μL).

### Extraction of Phlorotannins from *E*. *bicyclis*

Dried *E*. *bicyclis* (40 g) was extracted with 400 mL of 80% ethanol under continuous mixing at 37°C, overnight. After filtering, the sample was evaporated, dissolved in 20 mL of H_2_O, and extracted with CHCl_3_ followed by hexane. The aqueous phase was extracted with an equal volume of diethyl ether. The diethyl ether and aqueous phases were evaporated, dissolved with 1 mL H_2_O and used as phlorotannin fraction A and B, respectively.

### Purification of EHEP from the Digestive Fluid

All purification procedures were performed at 4°C. Frozen digestive fluid (200 mL) of *A*. *kurodai* was thawed and centrifuged at 20, 000 x *g* for 15 min. The supernatant was then fractionated with ammonium sulfate (0–35% saturation) and centrifuged at 20, 000 x *g* for 15 min. The precipitate formed was dissolved in 20 mM acetate buffer (pH 6.0), dialyzed against the same buffer at 4°C and centrifuged at 20,000 x *g* for 10 min. The supernatant obtained was concentrated by ultrafiltration, ammonium sulfate was added to produce a final 1 M concentration, and this solution was centrifuged at 20, 000 x *g* for 15 min. The precipitate formed was dissolved in 50 mM acetate (pH 6.0) containing 0.5 M ammonium sulfate, applied to a phenyl-Sepharose column (1.5 x 9.5 cm), and eluted with the same buffer. The fraction showing EHEP activity was pooled, concentrated by ultrafiltration and further purified by Sepharose S-100 gel filtration. The EHEP activity was eluted as a single peak containing a 25 kDa protein. Purity of the final preparation was checked by SDS-PAGE [45] and 2D-PAGE [46]. Isoelectric point of EHEP was determined by isoelectric focusing. After electrophoresis, the pH of the gel was measured after elution of 0.5 cm gel segment in 1.0 ml of H_2_O for 2 h.

### Purification of CDE from the Digestive Fluid

All purification procedures were performed at 4°C. Frozen digestive fluid (200 mL) of *A*. *kurodai* was thawed, centrifuged at 20, 000 x *g* for 15 min and the supernatant was fractionated with ammonium sulfate (35–60% saturation). After centrifugation at 20, 000 x *g* for 15 min, the precipitate obtained was dissolved in 20 mM acetate buffer (pH 6.0) and dialyzed against the same buffer at 4°C. The supernatant was applied to a CM-Sepharose column (2.5 × 20 cm) equilibrated with 20 mM acetate (pH 6.0) and eluted with the same buffer. Proteins unbound to the CM-Sepharose column were applied to a DEAE-Sepharose column (2.5 x 10 cm) and eluted with a linear gradient of NaCl (0–0.4 M). The fraction possessing CDE activity was pooled, concentrated and dialyzed against 20 mM acetate buffer, pH 5.0. The dialyzate was applied to a Mono S column and eluted with linear gradient of NaCl (0–0.2 M). The fraction showing CDE activity was eluted as a single peak and contained an 85 kDa protein.

### Analysis of Degradation Products by Thin Layer Chromatography

Thin layer chromatography (TLC) was performed in TLC Silica gel 60F plates (Merck KGaA, Darmstadt, Germany) to analyze the degradation products of laminaran, which were detected using orcinol-sulfuric acid, as previously described [47].

### Amino Acid Sequence Analysis

Purified EHEP was digested with pepsin in 20 mM acetate, or with lysyl endopeptidase in 50 mM Tris-HCl buffer (pH 8.8) at 37°C for 24 h. The digests were separated by SDS-PAGE [45] and electroblotted onto polyvinylidene fluoride (PVDF) membranes (Immobilon^™^, 0.45 mm, Millipore, Bedford, MA, USA) according to the manufacturer’s instructions. The fragments were stained with Ponceau 3R. The 16 and 6 kDa protein fragments produced by pepsin treatment, and the 21.5 kDa protein fragment produced by lysyl endopeptidase were used to determine the internal sequence of EHEP. Amino acid sequences were determined by an automated protein sequencer (Shimadzu PPSQ-10, Kyoto, Japan).

### Isolation of cDNA Encoding EHEP

Total RNA was isolated from the hepatopancreas of *A*. *kurodai*, and reverse-transcribed with Moloney murine leukemia virus reverse transcriptase using a random hexamer, as previously described [48]. To isolate the cDNA fragment encoding EHEP, we designed PCR primers (S4 and AS1) corresponding to the sequences of 6 kDa fragment (NNLATGIHP) generated from pepsin digestion of EHEP and 21.5 kDa fragment (NGAVATVM) generated from lysyl endopeptidase digestion. The sense (S4) and antisense (AS1) primer sequences used were 5’-AATGGCGCCGTTGCCACGGTGATG-3’ corresponding to NGAVATVM and 5’-GGGTGGATGCCGTTAGCCAGGTTGTT-3’ corresponding to NNLATGIHP, respectively. PCR was performed for 30 cycles comprising denaturation at 95°C for 1 min, annealing at 55°C for 30 s, followed by extension at 72°C for 1 min. The PCR products were then cloned into the pGEM-T Easy vector (Promega, Madison, WI, USA) and sequenced. Amplified cDNA (396 bp) was highly homologous with the corresponding region (amino acid residue number: 48–179) of chondroitin proteoglycan-2 like proteins (NCBI reference: XP_005103174) that were predicted from the *A*. *californica* genome sequence by automated computational analysis. The cDNA encoding the N-terminal side of EHEP was amplified by PCR, using primers corresponding to 5’-untranslated region (5’-GGCCCTCCCTTTGCTGGCAACATG, initial codon is underlined) of XP_005103174 (S4) and WGNGNFTHPYN (AS1). The sequences of the S1 and AS1 primers in the first PCR product were replaced with the corresponding sequence of the second PCR product in the composite sequence. The cDNAs for 5’- and 3’-terminal regions of mRNA were amplified using a First Choice RLM-RACE Kit (Ambion, Life Technologies Co, Carlsbad, CA, USA) according to the manufacturer’s protocol. The 5’-RACE inner and outer primers were, 5’-GTAGAAGTTGCCGTTGCCCCA-3’ (corresponding to nucleotide numbers 292–312) and 5’-GATGTACTTCCGGCAGTCGTA-3’ (115–135), respectively. The 3’-RACE inner and outer primers used were, 5’-CCCGACGGCTTCGACACCTACTGC-3’ (481–504) and 5’-GCCAACAACCTGGCCACCGGCATC-3’ (508–531), respectively. Primer sequences were replaced with the corresponding internal sequence of the PCR products in the composite sequence. The full-length EHEP cDNA amplified was cloned into the pGEM-T Easy vector (Promega).

### Analysis of Glycoprotein by Lectin Blot

The lectin blot of glycoprotein was performed using horseradish peroxidase-labeled lectins (ConA, LCA, PHA-E4, PNA, RCA120, and WGA) as described previously [18].

### Antimicrobial Activity Assay

*Escherichia coli* NITE Biological Resource Center (NBRC) 12713 and *Staphylococcus aureus* NBRC 12732 were cultivated (17 h, 37°C) in Luria-Bertani medium (LB broth, Lennox; Nacalai Tesque Inc., Kyoto, Japan). Bacterial suspensions (2.0 × 10^7^ cells/mL) were prepared in nutrient broth (NB, Becton, Dickinson and Company, Franklin Lakes, NJ, USA). An aliquot of bacterial suspension (0.05 mL) was mixed with 0.45 mL of NB containing 10 or 50 μg of EHEP and incubated at 37°C in a 48-well culture plate (AGC Tecno Glass Co., Ltd., Tokyo, Japan). As a control, bacteria were incubated in NB in the absence of EHEP. At appropriate times of incubation, the optical density of cultures was measured at 600 nm, in each of the 48-wells using a microplate reader (Infinite M200; Tecan Japan Co., Ltd, Kanagawa, Japan).

## Supporting Information

S1 Fig Purification of curdlan digestion enzyme (CDE) from the digestive fluid of *A*. *kurodai*. CDE was purified from the digestive fluid of *A*. *kurodai* by ammonium sulfate fractionation (35–60%), CM-Sepharose, phenyl-Sepharose, Sepacryl S-100 and Mono S chromatography as described in Materials and Methods. (A) Elution profile of CDE on Mono S chromatography. Analysis of eluate by SDS-PAGE is shown in the inset. Fractions indicated by the horizontal bar were pooled and used as purified CDE. (B) SDS-PAGE (10% gel) of the purified CDE (1 μg). (C) Alignment of the internal sequences of the purified enzyme with sequence of α-xylosidase A-like protein from *Aplysia californica* (NCBI Reference sequence XP_012938183), α-xylosidase from *Gallibacterium anatis* (NCBI Reference sequence WP_039080918, UniProt accession number A0A0A2XLL6), glucan 1,3-α-glucosidase from *Strongylocentrotus purpuratus* (NCBI Reference sequence XP_011667610, UniProt W4Y537) and α-glucosidase from *Clostridium symbiosum* (NCBI Reference sequence WP_003507443, UniProt G5FAK1). The three internal sequences of fragments (#20, 29 and 35) generated by lysyl endopeptidase digestion of the purified enzyme were determined as described in Materials and Methods. (D) Lectin blot of purified CDE. The purified enzyme was resolved by SDS-PAGE (10% gel) and detected using horseradish peroxidase labeled lectin (ConA and LCA), as described in Materials and Methods.
(TIF)

S2 Fig Mode of action of CDE on curdlan, laminarin and laminaran. (A) Digestion of curdlan and laminarin with CDE in the absence and presence of 210kDa BGL. Curdlan or laminarin, 0.5 ml of 0.5% (w/v) in Buffer A, was incubated with 0.05 U CDE and 0.2 U 210 kDa BGL, as indicated, at 37°C for 10, 30, 60, 120, and 180 min. Reaction products were analyzed by TLC. (B) Laminaran, 0.5 ml of 0.5% (w/v) in Buffer A, was incubated at 37°C for 1 h with 110 or 210 kDa BGL, as indicated in the absence and presence of 0.05 U of CDE. The glucose content in the reaction mixture was then determined. (C) Laminaran, 0.5 ml of 0.5% (w/v) in Buffer A, was incubated at 37°C for 1 h with 0.2 U of 110 and 210 kDa BGL in the absence or presence of 0.05 U CDE.
(TIF)
