# Supplementary figures and images for: Identification and Characterization of a 25 kDa Protein That Is Indispensable for the Efficient Saccharification of Eisenia bicyclis in the Digestive Fluid of Aplysia kurodai
Source: PLoS One. 2017 Jan 27;12(1):e0170669. doi: 10.1371/journal.pone.0170669 (PMC5271319; doi:10.1371/journal.pone.0170669)

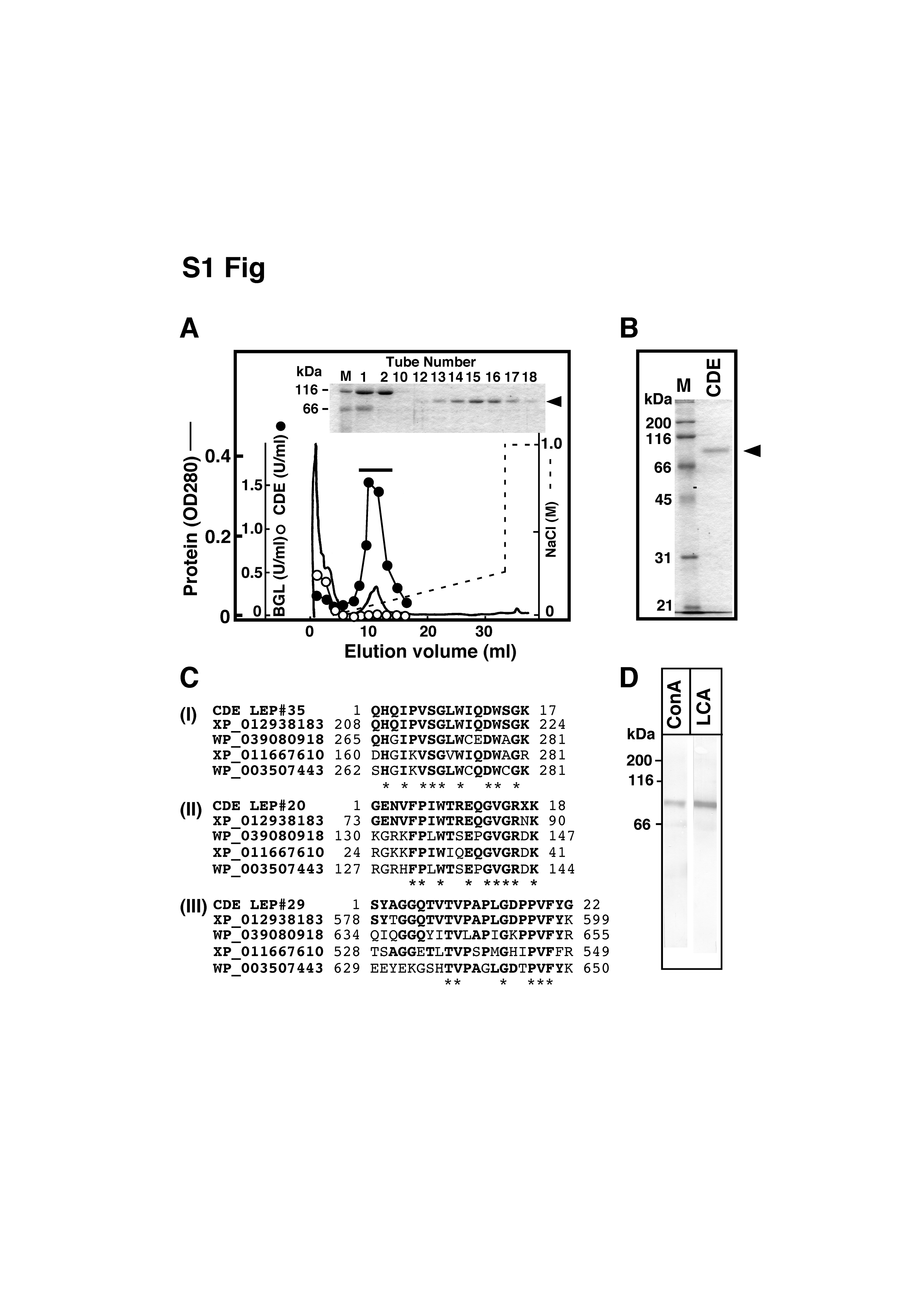

Supplement: S1 Fig — CDE was purified from the digestive fluid of A. kurodai by ammonium sulfate fractionation (35–60%), CM-Sepharose, phenyl-Sepharose, Sepacryl S-100 and Mono S chromatography as described in Materials and Methods. (A) Elution profile of CDE on Mono S chromatography. Analysis of eluate by SDS-PAGE is shown in the inset. Fractions indicated by the horizontal bar were pooled and used as purified CDE. (B) SDS-PAGE (10% gel) of the purified CDE (1 μg). (C) Alignment of the internal sequences of the purified enzyme with sequence of α-xylosidase A-like protein from Aplysia californica (NCBI Reference sequence XP_012938183), α-xylosidase from Gallibacterium anatis (NCBI Reference sequence WP_039080918, UniProt accession number A0A0A2XLL6), glucan 1,3-α-glucosidase from Strongylocentrotus purpuratus (NCBI Reference sequence XP_011667610, UniProt W4Y537) and α-glucosidase from Clostridium symbiosum (NCBI Reference sequence WP_003507443, UniProt G5FAK1). The three internal sequences of fragments (#20, 29 and 35) generated by lysyl endopeptidase digestion of the purified enzyme were determined as described in Materials and Methods. (D) Lectin blot of purified CDE. The purified enzyme was resolved by SDS-PAGE (10% gel) and detected using horseradish peroxidase labeled lectin (ConA and LCA), as described in Materials and Methods. (TIF) [file pone.0170669.s001.tif]

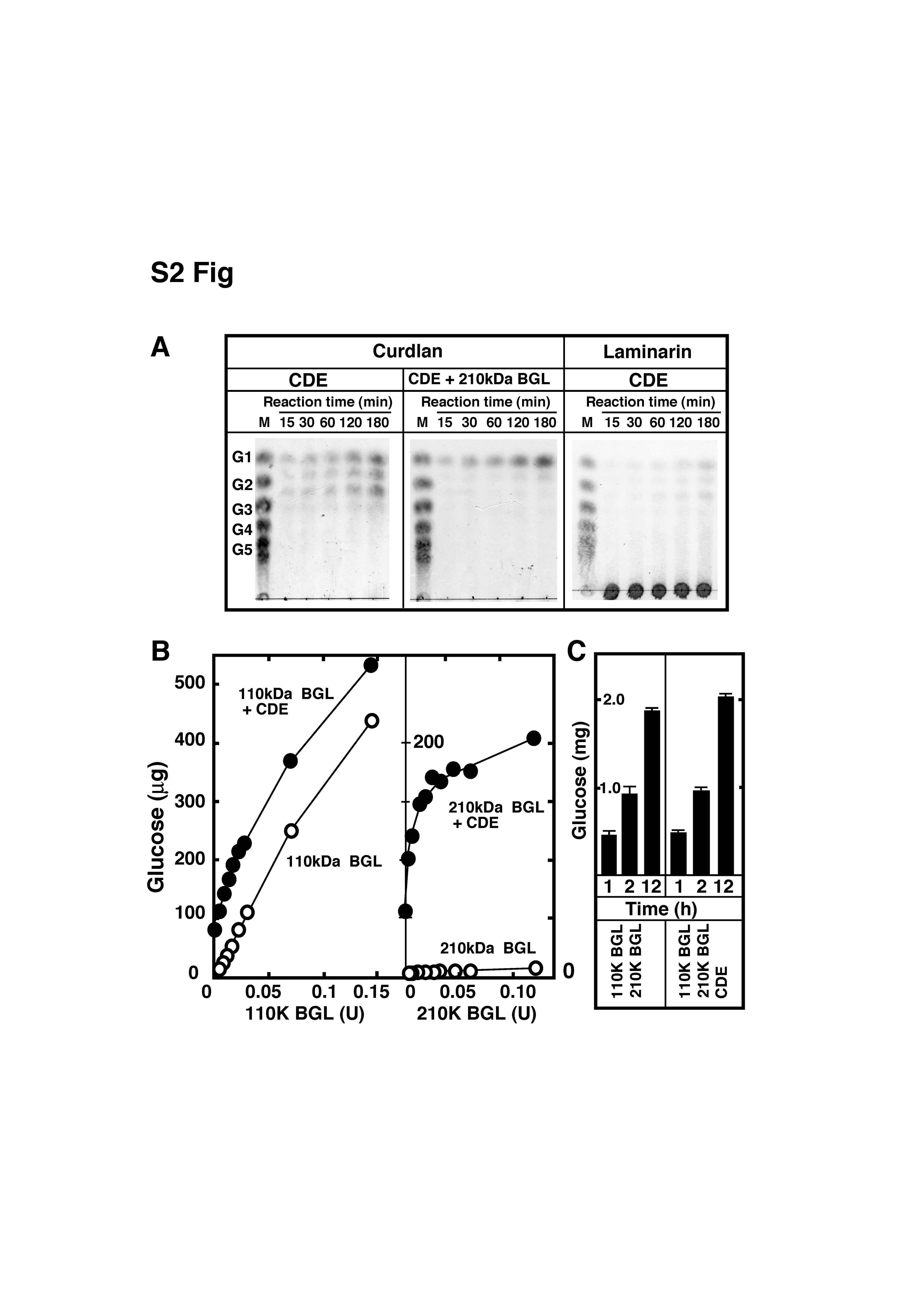

Supplement: S2 Fig — (A) Digestion of curdlan and laminarin with CDE in the absence and presence of 210kDa BGL. Curdlan or laminarin, 0.5 ml of 0.5% (w/v) in Buffer A, was incubated with 0.05 U CDE and 0.2 U 210 kDa BGL, as indicated, at 37°C for 10, 30, 60, 120, and 180 min. Reaction products were analyzed by TLC. (B) Laminaran, 0.5 ml of 0.5% (w/v) in Buffer A, was incubated at 37°C for 1 h with 110 or 210 kDa BGL, as indicated in the absence and presence of 0.05 U of CDE. The glucose content in the reaction mixture was then determined. (C) Laminaran, 0.5 ml of 0.5% (w/v) in Buffer A, was incubated at 37°C for 1 h with 0.2 U of 110 and 210 kDa BGL in the absence or presence of 0.05 U CDE. (TIF) [file pone.0170669.s002.tif]
